# Endovascular repair for arterio-venous fistula treatment: a case report

**DOI:** 10.1186/s44215-023-00088-5

**Published:** 2023-08-11

**Authors:** Jun Hayashi, Yuki Sekine, Shuji Toyama, Tetsuro Uchida, Tetsuo Watanabe

**Affiliations:** 1 Department of Cardiovascular Surgery, Sendai City Hospital, 1-1-1 Asuto Nagamachi, Taihaku Ward, Sendai City, Miyagi, 982-8502 Japan; 2 Second Department of Surgery, Faculty of Medicine, Yamagata University, 2‑2‑2 Iida‑Nishi, Yamagata, 990‑9585 Japan

**Keywords:** Arterio-venous fistula, Ruptured abdominal aortic aneurysm, Endovascular repair

## Abstract

**Background:**

Arterio-venous fistula is a rare clinical entity that occurs as a result of a ruptured abdominal aortic aneurysm into the inferior vena cava or iliac vein. Open surgery has been considered the treatment of choice for this condition; however, endovascular repair has become an evolving therapeutic option over the past 20 years. The endovascular treatment has contributed to a decrease in the perioperative mortality; nevertheless, some endovascular repair specific concern for residual fistula still remains.

**Case presentation:**

A 70-year-old man was referred to our department for an enlarged inferior vena cava without any complaint. Contrast-enhanced computed tomography demonstrated a 40-mm AAA and a 42-mm right saccular CIAA, with a fistula between the CIAA and left CIV. Endovascular aneurysm repair which focused on the exclusion of the right CIAA and prevention of endoleak formation was performed. To prevent type 2 endoleaks, which cause residual arterio-venous shunt flow, right internal iliac artery was embolized with a vascular plug prior to stent graft procedure. Successful aneurysm exclusion without residual arterio-venous shunt flow was confirmed with a final angiographic examination. The patient had an uneventful recovery, and computed tomography 1 month later showed no sign of persistent fistula.

**Conclusions:**

Although endovascular treatment for AVF has a negative possibility of residual shunt, stent graft with a focus on type 2 endoleak prevention can be an attractive treatment option, reducing the incidence of residual AVF.

## Background

Arterio-venous fistula (AVF) is a rare clinical entity, which is reported in 2–7% of ruptured abdominal aortic aneurysms (rAAAs) [1]. Eighty percent of patients with AVF exhibit erosion of the arterial wall and spontaneous shunting of the arterial blood into the adjacent vena cava or, less often, to the right iliac vein [2]. AVF results in major hemodynamic changes induced by the diversion of blood flow from the arterial to venous circulation. This process increases the venous pressure, thereby causing acute high-output heart failure [3]. Although urgent AVF repair is required to achieve hemostasis in the context of rAAAs, drastic hemodynamic changes should be avoided as they could cause lethal circulatory dysfunction [2].

AVF is classically treated via open surgical repair; however, there are reports of significant intraoperative bleeding and high morbidity and mortality rates associated with this intervention [3]. Over the past 20 years, endovascular aortic aneurysm repair (EVAR), a less invasive technique, has evolved into an alternative therapeutic option for patients with AVF [4]. EVAR provides an adequate, validated, and safe therapeutic option for AVF, with lower mortality rates [2]. However, several cases of postoperative endoleaks associated with persistent residual AVF have been reported [1]. Herein, we present a case of a ruptured right common iliac arterial aneurysm (CIAA) with a fistula to the left common iliac vein (CIV), which was managed by endovascular repair. The patient provided informed consent for the publication of this case report.

### Case presentation

**Fig. 1:**
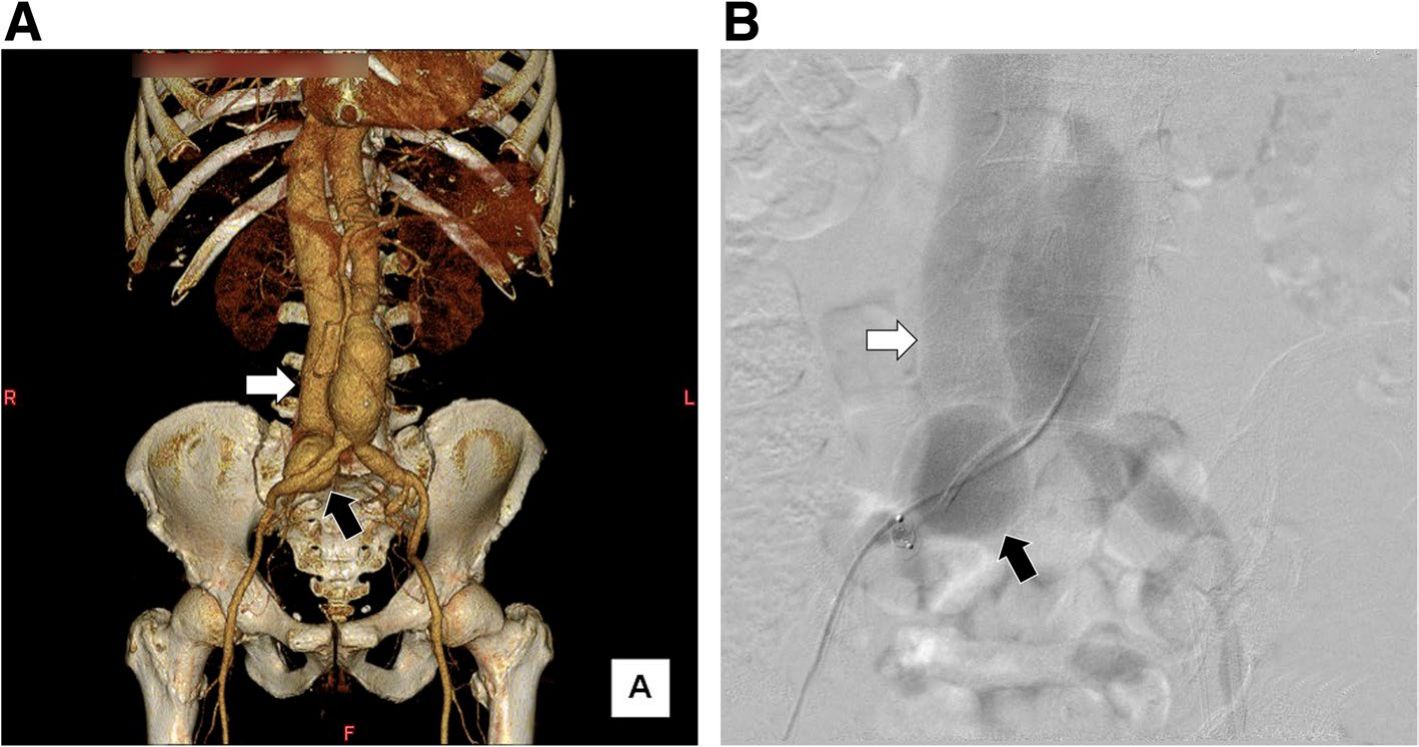
Preoperative three-dimensional reconstruction of computed tomography angiography and initial intraoperative angiography findings **A** Preoperative three-dimensional reconstruction of computed tomography angiography showing a 40-mm abdominal aortic aneurysm, 40-mm right common iliac arterial aneurysm with ilio-iliac fistula (black arrow), and severely dilated inferior vena cava (white arrow). **B** Intraoperative initial angiography showing a communication between the right iliac artery and left iliac vein (black arrow), as well as a severely dilated inferior vena cava (white arrow)

A 70-year-old man was referred to our department for an enlarged inferior vena cava with early phase contrast enhancement seen on computed tomography (CT). He previously underwent percutaneous coronary intervention (PCI) via the left radial artery for three-vessel disease 6 months prior to admission. He had repeated episodes of post PCI heart failure, for which he was admitted to the cardiology ward of our hospital. Contrast-enhanced CT demonstrated a 40-mm AAA and a 42-mm right saccular CIAA, with a fistula between the CIAA and left CIV (Fig. 1A). Physical examination revealed lower limb edema and a pulsatile abdominal mass with vascular thrill; however, no clinical signs of respiratory failure or laboratory findings indicative of liver or renal dysfunction noted on admission. The patient was stable based on clinical and radiographic findings and was consequently scheduled for elective endovascular repair.

The surgery was performed under general anesthesia using a bilateral femoral arterial approach (Fig. 1B). Preoperatively, a venous cannula was inserted into the inferior vena cava (IVC) via the left femoral vein to monitor intraoperative hemodynamic changes and ascertain complete fistula exclusion after endograft deployment. Preoperative CT and intraoperative angiography revealed that the origin of the AVF was the right CIAA. Thus, the operative strategy focused on the exclusion of the right CIAA and prevention of endoleak formation. The absence of arterial branches such as lumber artery or media sacral artery which could cause type 2 endoleaks was checked carefully on preoperative CT scan and intraoperative angiography. To prevent type 2 endoleaks from internal iliac arterial back-bleeding, right internal iliac artery embolization was performed using an Amplatzer Vascular Plug 2 (St. Jude Medical, Saint Paul, MN, USA). A straight graft (Excluder leg device; 16 $\times$ 12 $\times$ 120 mm; W. L. Gore & Assoc., Flagstaff, AZ, USA) was deployed from a sealable, non-dilated portion of the proximal right common iliac artery to the external iliac artery, effectively excluding the right CIAA and rendering it independent from the AAA. AAA exclusion was then performed following CIAA exclusion. Total aneurysm exclusion was achieved by deploying an Excluder main body stent graft from just below the renal artery to the right external and left common iliac arteries. Finally, a straight graft (Excluder leg device, 16 $\times$ 16 $\times$ 140 mm) was placed between the main body of the device and the first right iliac straight graft, thereby completing aneurysm exclusion.

**Fig. 2:**
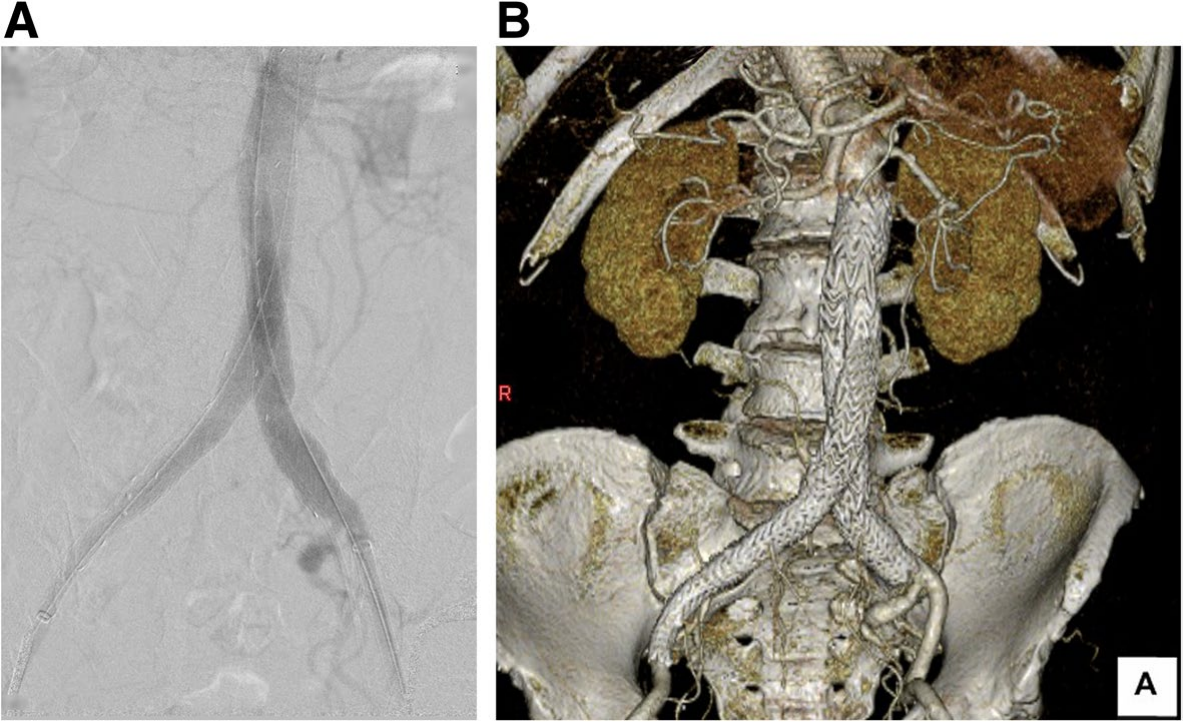
Final intraoperative angiography and postoperative three-dimensional reconstruction of computed tomography angiography findings **A** Intraoperative final angiography confirming the absence of persistent arterio-venous fistula and significant endoleak. **B** Postoperative three-dimensional reconstruction of computed tomography angiography performed one month after surgery showing complete aneurysm exclusion and fistula dissipation

As planned, successful aneurysm exclusion without residual arterio-venous shunt flow was confirmed using a final angiographic examination (Fig. 2A). Pre-deployment pressure monitoring at the IVC showed an arterial pulsatile wave pattern ranging from 10 to 11 mmHg. After stent graft deployment, the shape of the pressure curve changed into a continuous flow curve similar to the normal venous pressure curve, and the pressure decreased to 6 mmHg. Conversely, the systolic systemic pressure fluctuated, temporarily increasing to 131 mmHg and then dropping to 81 mmHg. However, the cardiac index was maintained, and hemodynamic parameters subsequently stabilized. The patient was asymptomatic during the postoperative period, and no significant complications were noted. The lower extremity edema gradually resolved after surgery. One-month postoperative CT confirmed complete aneurysm exclusion without type 2 endoleak (Fig. 2B). The CIAA and CIV were remodeled and shrunk, and the diameters were decreased from 42 to 29 mm and from 26 to 15 mm. There were no signs of persistent AVF or pulmonary embolization.

## Discussion

AVF was first reported in 1831, and the first successful open surgical repair was described by Cooley et al. in 1955 [5]. Despite high surgical morbidity and mortality rates, patients with AVF continued to undergo open surgical repair [3]. However, since the first endovascular AVF repair was described by Beveridge et al. in 1998 [4], an alternative intervention has been recognized. Several studies on endovascular AVF treatment have since been published. In 2021, Borghese et al. conducted a systematic review on 46 and 33 patients who underwent open and endovascular repairs, respectively, between 1999 and 2018. The 30-day mortality rates in the open and endovascular repair groups were 10.8% and 3.0%, respectively [3]. These results supported the use of the endovascular method for AVF repair to reduce the risk of complications and death.

Although endovascular surgery seems to be a good therapeutic option with low risks of morbidity and mortality, it is associated with several specific concerns, such as the possibility of residual AVF. Melas et al. described a case of a patient with residual AVF after EVAR. Although the patient was stable 6 months after surgery, the AVF increased in size and the patient presented with signs of progressing heart failure [6]. Most of these persistent fistulas are considered to be associated with type 2 endoleaks, which may occur in up to 50% of patients treated by EVAR [1]. Further, the assessment and management of persistent AVF due to type 2 endoleak after endovascular treatment is controversial. Minimal shunt flow may be clinically insignificant; however, a significant shunt flow can cause heart failure and require therapeutic intervention. Nakad et al. conducted a systematic review wherein they reported that 11% of patients with persistent AVF due to type 2 endoleak required reintervention [7]. Moreover, some authors recommend venous stent-graft insertion in patients with the abovementioned condition. Conversely, considering that 89% of patients with persistent AVF would not require additional intervention, a small residual shunt would not always yield adverse effects [8]. Wang et al. showed that residual AVF causes a pressure gradient because of its connection with the low-pressure venous system, resulting in decreased in-sac pressure [8]. Therefore, the aneurysm sac and fistula orifice may shrink gradually under the low in-sac pressure. Luijtgaarden et al. also emphasized that type 2 endoleaks could resolve spontaneously [9]. They presented a case of a patient with aneurysm sac shrinkage resulting from residual AVF at 1 year after EVAR. A comprehensive review of the relevant published studies was undertaken and the results were shown in Table 1, including the cases referred to here [7, 9–42].

**Table 1:** Summary of AVF cases treated with EVAR

Variables	*N* = 43
Mean age, range	67.9 [41–89]
Male sex, *n* (%)	42 (98)
Symptom, *n* (%)	
Abdominal pain/back pain	23 (54)
Limb swelling	15 (35)
Dyspnea	10 (23)
Pulmonary embolization	1 (2.3)
Post-operative complications, *n* (%)	
Acute renal failure	4 (9.3)
Type 2 endoleak with residual AVF^a^	3 (7.0)
Type 2 endoleak without residual AVF	3 (7.0)
Pulmonary embolization	2 (4.7)
Spinal cord injury	0 (0)
Perioperative mortality^b^	4 (9.3)
Late mortality	2 (4.7)

^a^One patient was managed conservatively, one patient was performed transcaval coil embolization, and the other one was performed percutaneous closure with the muscular VSD occluder

^b^The causes of death were sepsis in 2 patients, ischemic colitis in 1 patient, and multiple organ failure in 1 patient. *AVF* Arterio-venous fistula, *EVAR* endovascular aortic repair

Although most persistent trivial fistula shunts do not present with severe symptoms or resolve spontaneously, Fujimiya et al. emphasized the risks of AVF-specific complications following EVAR [43]. They presented a case of a patient with a type 2 endoleak from the inferior mesenteric artery (IMA), which coexisted with a persistent AVF after EVAR. Four months after EVAR for AVF, there was an occurrence of spontaneous AVF obstruction and subsequent rapid aneurysm sac dilatation due to the type 2 endoleak. Direct ligation of the IMA via median laparotomy was required, highlighting that EVAR alone is insufficient for AVF treatment where IMA or large lumbar arteries are involved. In our case, the AVF involved the CIAA and had no arterial branches. Thus, the probability of developing a type 2 endoleak was considerably low, and we determined that treatment using a stent graft without embolization except for the internal iliac artery was possible, which was achieved successfully. However, in cases where the aneurysm has arterial branches that can cause endoleaks, additional procedures such as embolization should be performed.

Although endovascular treatment has specific concerns, it is widely recognized as a less invasive alternative to open surgery. In contrast to non-ruptured AAA, type 2 endoleak can occur residual AVF, which can affect patient outcomes. Thus, reliable coiling should be applied for large IMA or lumbar arteries to prevent type 2 endoleak occurrence. We believe that endovascular surgery with a focus on type 2 endoleak prevention would reduce the incidence of residual AVF, thus improving the outcomes of patients with AVF.

We present the successful repair of a ruptured right CIAA with a fistula to the left CIV using an endovascular graft. We believe the endovascular repair can be an attractive treatment option for the AVF, provided the type 2 endoleak is effectively prevented.

## Data Availability

All data supporting the conclusions of this article are included within the published article.

## Abbreviations

AVF: Arterio-venous fistula
AAA: Abdominal aortic aneurysms
EVAR: Endovascular aortic aneurysm repair
CIAA: Common iliac arterial aneurysm
CIV: Common iliac vein
CT: Computed tomography
PCI: Percutaneous coronary intervention
IVC: Inferior vena cava
IMA: Inferior mesenteric artery

## References

[1] Orion KC, Beaulieu RJ, Black JH. Aortocaval fistula: is endovascular repair the preferred solution? Ann Vasc Surg. 2016;31:221e8. 26597238 10.1016/j.avsg.2015.09.006 PMC4860718

[2] Torrealba JI, Vargas JF, Mertens RA, Valdes FJ, Marine LA, Bergoeing MP. Endovascular management of a ruptured iliac aneurysm with an inferior vena cava fistula. Vasc Endovascular Surg. 2020;54:638–42. 32662320 10.1177/1538574420939724

[3] Borghese O, Pisani A, Sbenaglia G, Giudice R. Open surgery and endovascular techniques in treatment of acute abdominal arteriovenous fistulas. Ann Vasc Surg. 2019;61:427–33. 31207397 10.1016/j.avsg.2019.03.041

[4] Beveridge CJ, Pleass HC, Chamberlain J, Wyatt MG, Rose JD. Aortoiliac aneurysm with arteriocaval fistula treated by a bifurcated endovascular stent-graft. Cardiovasc Intervent Radiol. 1998;21:244–6. 9626443 10.1007/s002709900253

[5] Javid H, Dye WS, Grove WJ, Julian OC. Resection of ruptured aneurysms of abdominal aorta. Ann Surg. 1955;142:623. 10.1097/00000658-195510000-00006 PMC1465188 13259423

[6] Melas N, Saratzis A, Saratzis N, Lazaridis I, Kiskinis D. Inferior vena cava stent-graft placement to treat endoleak associated with an aortocaval fistula. J Endovasc Ther. 2011;18:250–4. 21521067 10.1583/10-3296.1

[7] Nakad G, AbiChedid G, Osman R. Endovascular treatment of major abdominal arteriovenous fistulas: a systematic review. Vasc Endovascular Surg. 2014;48:388–95. 24973241 10.1177/1538574414540485

[8] Wang T, Zhao J, Yuan D. Endovascular treatment of an ilio-iliac arteriovenous fistula accompanied by venous thromboembolism presenting with multiple organ failure - a case report and literature review. Vascular. 2021:1708538121996576. 10.1177/1708538121996576 33663299

[9] van de Luijtgaarden KM, Bastos Goncalves F, Rouwet EV, Hendriks JM, Ten Raa S, Verhagen HJ. Conservative management of persistent aortocaval fistula after endovascular aortic repair. J Vasc Surg. 2013;58:1080–3. 23478500 10.1016/j.jvs.2012.10.138

[10] Lau LL, O’Reilly MJ, Johnston LC, Lee B. Endovascular stentgraft repair of primary aortocaval fistula with an abdominal aortoiliac aneurysm. J Vasc Surg. 2001;33:425e8. 11174799 10.1067/mva.2001.111485

[11] Shah TR, Parikh P, Borkon M, Mocharla R, Lonier J, Rosenzweig BP, et al. Endovascular repair of contained abdominal aortic aneurysm rupture with aortocaval fistula presenting with high-output heart failure. Vasc Endovascular Surg. 2013;47:51e6. 23051851 10.1177/1538574412462633

[12] Umscheid T, Stelter WJ. Endovascular treatment of an aortic aneurysm ruptured into the inferior vena cava. J Endovasc Ther. 2000;7:31e5. 10772746 10.1177/152660280000700105

[13] Rapacciuolo A, De Angelis MC, di Pietro E, Puglia R, Di Tommaso E, Ruggiero D, et al. Percutaneous treatment of an aorto-caval fistula in an old high risk patient. BMC Surg. 2012;12(Suppl 1):S32. 23173555 10.1186/1471-2482-12-S1-S32 PMC3499279

[14] ElKassaby M, Alawy M, Zaki M, Hynes N, Tawfick W, Sultan S. Total endovascular management of ruptured aortocaval fistula: technical challenges and case report. Vascular. 2014;22:306e9. 24000081 10.1177/1708538113499018

[15] Silveira PG, Cunha JR, Barbosa Lima GB, Franklin RN, Bortoluzzi CT, Galego GN. Endovascular treatment of ruptured abdominal aortic aneurysm with aortocaval fistula based on aortic and inferior vena cava stentgraft placement. Ann Vasc Surg. 2014;28:1933. 10.1016/j.avsg.2014.06.073 25017775

[16] LaBarbera M, Nathanson D, Hui P. Percutaneous closure of aortocaval fistula using the amplatzer muscular VSD occlude. J Invasive Cardiol. 2011;23:343e4. 21828399

[17] Guzzardi G, Fossaceca R, Divenuto I, Musiani A, Brustia P, Carriero A. Endovascular treatment of ruptured abdominal aortic aneurysm with aortocaval fistula. Cardiovasc Intervent Radiol. 2010;33:853e6. 19572169 10.1007/s00270-009-9640-5

[18] Leon LR Jr, Arslan B, Ley E, Labropoulos N. Endovascular therapy of spontaneous aortocaval fistulae associated with abdominal aortic aneurysms. Vascular. 2007;15:35e40. 17382053 10.2310/6670.2007.00011

[19] Kwon SH, Oh JH, Park SJ, Park HC. Endovascular repair of a spontaneous right common iliac arterydinferior vena cava fistula due to infrarenal aortoiliac aneurysm. Vasc Endovascular Surg. 2008;42:279e83. 18258725 10.1177/1538574407312649

[20] Juszkat R, Pukacki F, Zarzecka A, Kulesza J, Majewski W. Endovascular treatment of ruptured abdominal aneurysm into the inferior vena cava in patient after stent graft placement. Cardiovasc Intervent Radiol. 2009;32:776e80. 19267154 10.1007/s00270-009-9525-7

[21] Yuminaga Y, Mohabbat W, Nguyen T, Thompson AM, Fisher CM. Simultaneous endovascular repair of an iatrogenic carotid-jugular fistula and a large iliocaval fistula presenting with multiorgan failure: a case report. J Med Case Rep. 2012;6:33. 22273992 10.1186/1752-1947-6-33 PMC3277481

[22] Akwei S, Altaf N, Tennant W, MacSweeney S, Braithwaite B. Emergency endovascular repair of aortocaval fistulada single center experience. Vasc Endovascular Surg. 2011;45:442e6. 21571773 10.1177/1538574411407087

[23] Sultan S, Zaki M, Alawy M, ElKassaby M. Aortic and inferior vena cava bifurcated stent graft application in the endovascular management of a ruptured abdominal aortic aneurysm with an aortocaval fistula. J Vasc Surg. 2014;60:1665e6. 25454109 10.1016/j.jvs.2014.07.095

[24] Bernstein J, Jimenez JC. Inferior vena cava thrombosis following endovascular repair of acute aortocaval fistula: a word of caution. Vasc Endovascular Surg. 2013;47:467e9. 23709272 10.1177/1538574413490839

[25] Hetzel G, Gabriel P, Rompel O, Ritter W, Raithel D. Aortocaval fistula after stent-graft repair. J Endovasc Ther. 2006;13:117e20. 16445316 10.1583/05-1558MR.1

[26] Wang T, Huang B, Zhao J, Yang Y, Yuan D. Aortocaval fistula resulting from rupture of abdominal aortic dissecting aneurysm treated by delayed endovascular repair: a case report. Medicine. 2016;95:e3570. 27149481 10.1097/MD.0000000000003570 PMC4863798

[27] Fukuda I, Minakawa M, Fukui K, Suzuki Y. Management of an aorto-caval fistula from a ruptured aortic false aneurysm using a covered stent graft. Interact Cardiovasc Thorac Surg. 2007;6:682e4. 17670736 10.1510/icvts.2007.153064

[28] Kopp R, Weidenhagen R, Hoffmann R, Waggershauser T, Meimarakis G, Andrassy J, et al. Immediate endovascular treatment of an aortoiliac aneurysm ruptured into the inferior vena cava. Ann Vasc Surg. 2006;20:525e8. 16732443 10.1007/s10016-006-9061-8

[29] Wang Y, Yu W, Li Y, Wang H. Emergent endovascular repair of challenging aortocaval fistula with hostile anatomy. Vasc Endovascular Surg. 2017;51:255e60. 28486843 10.1177/1538574417701323

[30] Desai R, Akbashev M, Rubinsztain L, Kacharava A. The physical examination does matter: a case of spontaneous aortocaval fistula. Cureus. 2017;9:e1459. 28929042 10.7759/cureus.1459 PMC5593748

[31] Godart F, Haulon S, Houmany M, Francart C, Breviere GM, Rey C, et al. Transcatheter closure of aortocaval fistula with the amplatzer duct occluder. J Endovasc Ther. 2005;12:134e7. 15683265 10.1583/04-1332.1

[32] Vandereyken F, Schwagten V, Hertoghs M, Beaucourt L, D’Archambeau O, Hendriks J. Spontaneous ilio-iliac arteriovenous fistula due to an iliac artery aneurysm: a case-report. Acta Chir Belg. 2012;112:164e6. 22571082 10.1080/00015458.2012.11680817

[33] Duxbury MS, Wells IP, Roobottom C, Lambert AW. Endovascular repair of spontaneous no-aneurysmal aortocaval fistula. Eur J Vasc Endovasc Surg. 2002;24:276e8. 12217293 10.1053/ejvs.2002.1708

[34] Melas N, Saratzis A, Abbas A, Sarris K, Saratzis N, Lazaridis I, et al. Endovascular repair of a spontaneous ilio-iliac fistula presenting as pulmonary embolism. Vasa. 2011;40:246e50. 21638254 10.1024/0301-1526/a000100

[35] Tasaka S, Nomura T, Shoji K, Wada N. Aortocaval fistula noticed due to general malaise. Intern Med. 2022. 10.2169/internalmedicine.0608-22. 36130894 10.2169/internalmedicine.0608-22 PMC10208775

[36] de Oliveira C, Schmid BP, Molinari GJDP, Guillaumon AT. Abdominal aortic aneurysms ruptured to the vena cava: a case series and literature review. J Vasc Bras. 2021;20:e20200174. 34093691 10.1590/1677-5449.200174 PMC8147892

[37] Esmat HA, Naseri MW. Endovascular management of aortocaval fistula complicating abdominal aortic aneurysm presenting as an acute renal failure. Ann Med Surg (Lond). 2021;62:477–80. 33604036 10.1016/j.amsu.2021.01.090 PMC7873572

[38] Ascoli Marchetti A, Oddi FM, Diotallevi N, Battistini M, Ippoliti A. An unusual complication after endovascular aneurysm repair for giant abdominal aortic aneurysm with aortocaval fistula: High bilirubin levels. SAGE Open Med Case Rep. 2020;8:2050313X20984322. 10.1177/2050313X20984322 PMC7768567 33489236

[39] Treil L, Chakfé N. Colour 3D-reconstructed computed tomography angiography delineates blood flow through aortocaval fistula pre- and post-EVAR. Eur J Vasc Endovasc Surg. 2021;61:339. 33199213 10.1016/j.ejvs.2020.09.015

[40] De Boodt H, Pardon HE, Gellens P, Maleux G, Marrannes J. Balloon-assisted transcaval embolization of a type II endoleak associated with an aortocaval fistula after endovascular aortic repair. J Vasc Surg Cases Innov Tech. 2020;6:447–9. 32875178 10.1016/j.jvscit.2020.06.014 PMC7451716

[41] Greenfield S, Martin G, Malina M, Theivacumar NS. Aortocaval fistula, a potentially favourable complication of abdominal aortic aneurysm rupture in endovascular repair. Ann R Coll Surg Engl. 2020;102:e180–2. 32436721 10.1308/rcsann.2020.0090 PMC7538739

[42] Dabbouseh NM, Mason PJ, Patel PJ, Rossi PJ. Endovascular repair of delayed traumatic aortocaval fistula. J Vasc Surg Cases Innov Tech. 2019;5:467–71. 31763500 10.1016/j.jvscit.2019.06.012 PMC6859229

[43] Fujimiya T, Seto Y, Ishida K, Takase S, Satokawa H, Yokoyama H. Impending rupture of abdominal aortic aneurysm due to spontaneous obstruction of aortocaval fistula after endovascular abdominal aortic aneurysm repair. J Vasc Surg Cases Innov Tech. 2021;7:219–22. 33997557 10.1016/j.jvscit.2021.02.002 PMC8095077

